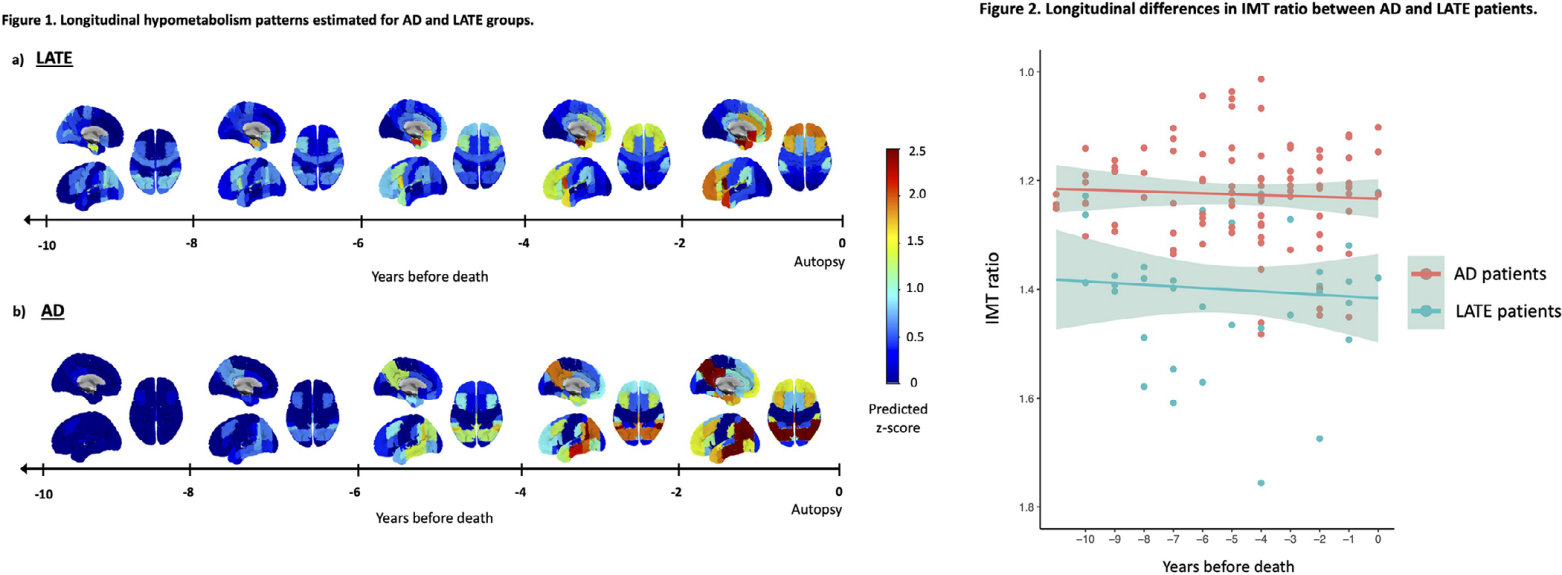# Longitudinal evolution of brain hypometabolism patterns in autopsy‐confirmed AD and LATE

**DOI:** 10.1002/alz.088877

**Published:** 2025-01-09

**Authors:** Miguel Labrador‐Espinosa, Jesús Silva‐Rodríguez, Alexis Moscoso, Pascual Sanchez‐Juan, Michael Schöll, Michel J. Grothe

**Affiliations:** ^1^ Wallenberg Centre for Molecular and Translational Medicine, University of Gothenburg, Gothenburg Sweden; ^2^ Reina Sofia Alzheimer Center, CIEN Foundation, ISCIII, Madrid, Madrid Spain; ^3^ Reina Sofia Alzheimer Centre, CIEN Foundation, ISCIII, Madrid Spain

## Abstract

**Background:**

Limbic‐predominant age‐related TDP‐43 encephalopathy (LATE) can underlie clinical presentations mimicking Alzheimer's disease (AD). Recent imaging‐pathological studies have shown that LATE associates with a specific temporo‐limbic FDG‐PET signature that differs from the typical temporo‐parietal pattern of hypometabolism in AD and may be of clinical utility for differential dementia diagnosis. Little is known about the temporal evolution of the respective hypometabolic patterns from early disease stages. We investigated the temporal evolution of LATE‐ and AD‐specific hypometabolism patterns using longitudinal ante‐mortem FDG‐PET data from autopsy‐confirmed cases.

**Method:**

Serial ante‐mortem FDG‐PET scans acquired in an interval of ‐11 to 0 years before death (mean: ‐4.8±2.9 years; median of 3 scans/subject) were analysed from 30 autopsy‐confirmed AD patients and 10 LATE patients enrolled in ADNI. FDG‐PET images were spatially normalized to MNI space and standard uptake value ratios (pons reference) were calculated across 52 brain regions defined in the Harvard‐Oxford atlas. Region‐wise z‐scores were calculated to quantify hypometabolism relative to a healthy elderly control group (N=179). Group‐wise longitudinal metabolic decline of brain regions was calculated using linear mixed‐effects models. Predicted z‐scores were estimated in 2‐year steps over a 10‐year interval from the time of death and referenced to approximate onset of first memory problems. In addition, we evaluated the longitudinal evolution of the inferior‐to‐medial temporal ratio (IMTr) as a previously proposed differential diagnostic imaging marker.

**Result:**

In AD, first pronounced hypometabolism (z>1) was observed in the posterior cingulate cortex and precuneus, approximately 6 years before death (5.5±4.5 years after estimated symptom onset) and progressively affecting additional temporal and lateral parietal brain regions (Fig‐1a). By contrast, first hypometabolism in LATE was observed in the hippocampus/parahippocampal cortex as early as 10 years before death, coinciding with the estimated onset of cognitive symptoms (0.1±3.3 years) and progressively including anterior temporal, subcallosal, and frontal brain regions (Fig‐1b). Interestingly, the IMT ratio showed a constant difference between AD and LATE patients across the entire observation period, including earliest disease stages (t=3.04, p=0.005)(Figure 2).

**Conclusion:**

LATE and AD show markedly different origins and temporal trajectories of regional brain hypometabolism, indicating that FDG‐PET may differentiate between these pathologies even at early disease stages.